# Clinical impact of bacterial contamination of perfusion fluid in kidney transplantation

**DOI:** 10.1186/s40064-015-1658-3

**Published:** 2016-01-04

**Authors:** A. Ranghino, D. Diena, F. Simonato, M. Messina, M. Burdese, V. Piraina, F. Fop, G. P. Segoloni, L. Biancone

**Affiliations:** Renal Transplantation Center “A. Vercellone”, Division of Nephrology Dialysis and Transplantation, Department of Medical Sciences, Città della Salute e della Scienza Hospital and University of Torino, Corso Dogliotti 14, 10126 Turin, Italy; Nephrology and Dialysis Unit, University of Magna Grecia, Catanzaro, Italy

**Keywords:** Perfusion fluid, Bacterial contamination, Kidney transplant, Preemptive therapy, Perioperative antibiotic prophylaxis

## Abstract

Contamination of perfusion fluid (PF) could lead to serious infections in kidney transplant recipients. Preemptive therapy (PE-T) in case of yeast contamination of PF is mandatory. The usefulness of PE-T in presence of bacteria remains unclear. In this study we evaluated the incidence of PF bacterial contamination and the impact of PE-T on clinical outcome. Microbiological data of 290 PF and clinical data of the corresponding recipients collected in our hospital from January 2010 and December 2012 were analyzed. Recipients with bacterial contaminated PF (101) were divided in 3 groups: group 1 (n = 52) PE-T treated bacteria resistant to perioperative antibiotic prophylaxis (PAP), group 2 (n = 28) bacteria sensitive to PAP, group 3 (n = 21) PE-T-untreated bacteria resistant to PAP. Incidence of positive PF was 34.8 %, 50.4 % staphylococci, 9.9 % *C. albicans*. No significant differences in the rate of PF-related infections between the three groups were found. In conclusion, although PF contamination is frequent, the incidence of PF-related infections is very low. In addition, in this study PE-T did not help to reduce the rate of PF-related infection suggesting that a resonable reduction in the use of antibiotic terapy could be made. However, waiting for largest and prospective clinical trials to confirm our findings, a closely clinical and microbiologic monitoring of the recipient is highly recommended in case of PF contamination.

## Background

Infectious complications constitute the major cause of morbidity and mortality especially in the post-operative period in patients undergoing solid organ transplantation (Rubin 1993; Fishman and Rubin 1998). It is known that infections in transplanted patients might develop as consequences of the immunosuppression-induced impairment of the inflammatory response and of the exposure to hospital- and community-acquired pathogens such as viruses, bacteria and yeast. Moreover, infections can be transmitted from the donor through the contaminated graft (Grossi et al. 2009; Fisher et al. 2009). Beside donor infection, graft contamination can occur during several stages in the process of deceased donor transplantation including the contamination of the perfusion fluid (PF) used to perfuse the kidney following donor nephrectomy (Fisher et al. 2009; Wakelin et al. 2005). PF contamination can be due to exogenous pathogens derived from handling of the organs and the subsequently exposure to contaminants during the procurement process especially in the multi-organ procurement (Wakelin et al. 2005). The incidence of PF contamination reported in literature ranges from 7 to 38.7 % and the pathogens isolated include Gram positive and negative bacteria and yeast (Anderson et al. 1978; Benoit et al. 1988; Mora et al. 1991; Sharma et al. 2005; Sauget et al. 2011; Veroux et al. 2010). It is known that contamination of PF could lead to serious infections such as mycotic arteritis and/or aneurysm due to the destruction of the vascular structures in consequence of the intense inflammatory response induced by bacteria or yeast infection (Fishman and Rubin 1998; Mai et al. 2006; Matignon et al. 2008; Albano et al. 2009; Gari-Tussaint et al. 2004). Nevertheless, except in the case of PF contaminated by *Candida sp* where the general consensus is to establish a preemptive therapy (PE-T) (Matignon et al. 2008), it remains unclear whether an antibiotic PE-T might help to avoid infections in presence of PF contamination by bacteria.

In this retrospective study the rate of PF contamination, the incidence of PF-related infections in the kidney transplanted recipients and the impact of the antibiotic PE-T in presence of PF bacterial contamination on clinical outcome were analyzed.

## Methods

In this study, we carried out a retrospective analysis of 290 PF samples and clinical data of patients receiving a kidney transplant from deceased multi-organ donors at our University hospital between January 2010 and December 2012. All recipients were followed up for at least 2 months post-transplantation. Recipient age, gender, immunosuppressive therapy, post-transplant use of antibiotic, donor age and cold ischemia time were collected. In addition, clinical and laboratory data such as body temperature, leukocyte count and C-reactive protein were collected daily for each recipient until discharged from the hospital and, subsequently, weekly. A careful physical examination was undertaken every day and routinely urine cultures were performed every weeks after transplantation for the first 2 months and subsequently every 2 weeks until the third month after transplantation.

PF samples obtained from the bag containing the kidney were collected before the back-table dissection of the graft prior to implantation. Each PF sample was inoculated both in aerobic and anaerobic blood culture bottles (BacT/Alert^®^, BioMerieux) and subjected to microbiological analysis.

All transplant recipients received prophylactic broad-spectrum antibiotics (PAP) intravenously (*iv*) at surgery and at least for nine days after transplantation. From January 2010 to December 2011 a PE-T was administered *iv* in the case of contaminated PF either by bacteria or yeast according to the antibiotic sensitivities. From January 2012, in order to reduce the potential acquirement of antimicrobial drug resistance and the development of multiresistant bacteria strains such as carbapenem-resistant Gram-negative pathogens, a PE-T was not administered in case of PF contaminated by bacteria. Nevertheless, the clinical and the laboratory markers of infection in the recipients receiving a graft with a contaminated PF were strictly monitored for at least 20 days post-transplantation or as long as the patient was discharged from the hospital.

In presence of yeast strain isolation in the PF, antifungal PE-T with fluconazole was started promptly according to the antimicogram. In the case of yeast contamination of the PF, as demonstrated by a bacterioscopic analysis, an antifungal PE-T with caspofungin was established prior of the culture isolation of the yeast strain and subsequently modified according to antifungal sensitivities. Caspofungin treatment was used as empiric therapy in the case of yeast contamination of PF for the follow reasons: (i) some yeast are resistant to fluconazole (*Candida glabrata* and *Candida kreusi*); (ii) in this setting the risk to develop vessels infections, such as mycotic arteritis, is higher compared to urinary infection. Since caspofungin reach high plasma concentration, this risk can be reduced. Recipients with yeast positive PF underwent blood, urine and drainage fluid cultures together with the daily screening of the inflammatory markers. In addition a Doppler ultrasound was performed at the time of diagnosis of contaminated PF by fungi to check vascular anastomoses and repeated according to clinician’s assumption.

All recipients received co-trimoxazole for *Pneumocystic jiroveci* prophylaxis starting from 10 days post-transplantation.

Statistical analysis was performed with SPSS (SPSS Inc. Chicago IL, vers. 20). Continuous variables are presented as mean ± standard deviation or as median [min–max], according to their distribution. The difference between groups was analyzed, respectively, with *t* test or Anova and Bonferroni and with Kruskal–Wallis test. Categorical variables are presented as fraction and Pearson’s *χ*^2^ or, for small samples, Fisher’s exact test was employed to compare groups. Significance level for all tests was set at 0.05.

## Results

The baseline characteristics of the 290 recipients and the transplant data are reported in Table 1. 101 out of 290 samples of PF collected were contaminated with one or more organisms resulting in an overall incidence rate of 34.8 % over the 3 years period. The organisms isolated were mainly staphylococci 51/101 (50.4 %), *Staphylococcus aureus* was detected in 4 PFs (3.9 %). *Pseudomonas aeruginosa* and *Candida albicans* were isolated in 5 (4.9 %) and in 10 (9.9 %) PF samples, respectively, Table 2. There were no significant differences in the immunosuppressive therapy between recipients who developed a PF-related urinary infection and those who did not. None of the 10 recipients who received a graft with a contaminated PF by *Candida albicans* developed any signs related to fungal infection such as renal arteritis and/or arterial aneurisms. Blood, urine and drainage fluid cultures in these patients were negative. PAP was administered to all transplanted recipients at surgery and for a mean duration of 12.5 ± 3.4 days post-transplantation as listed in Table 3. To test the impact of the antibiotic PE-T on the rate of infectious complications in transplanted patients receiving a graft with a positive PF for bacteria we compared the following three groups of patients: group 1 (n = 52) included patients with a pathogen in PF resistant to PAP that were subjected to PE-T, group 2 (n = 28) included recipients with contaminated PF by an organism sensitive to the PAP and who did not received PE-T and, group 3 (n = 21) included recipients with a pathogen in PF resistant to PAP who did not received PE-T. The mean duration of PE-T was 11.5 ± 4 days and the antibiotic regimens used are reported in Table 4. In the group 1, PE-T was started soon after the isolation of the pathogen in the perfusion fluid (7.1 ± 1.4 days post-transplantation). 4/101 (3.9 %) of the transplant recipients who received a kidney with a contaminated PF developed a urinary infection, 1 (*E. coli*) in group 1 and 3 (2 *E. coli*, 1 *Staphylococcus epidermidis*) in group 2, respectively. None of the patients in group 3 developed infections. No significant differences in the distribution of the organism isolated and in the rate of PF-related infections were found between the three groups. Noteworthy, among these bacteria, only one of the *E. coli* isolated in group 2, 9 days after transplantation, displayed the same antibiotic sensitivity of the organism isolated in the PF suggesting that both samples could be contaminated by the same pathogen. The remaining 3 bacteria isolated in the urine did not seem to be the same strain found in PF on the basis of: (i) higher distance from transplantation (mean: 19 days), and (ii) different antibiotic sensitivity. In order to evaluate whether some clinical and laboratory markers of inflammation could help to discriminate the recipients with contaminated PF who are at risk of developing PF-related infections, we analyzed the following data: body temperature, leukocyte count, and C-reactive protein levels at the time of reporting the positivity of the PF cultures. No significant differences in such markers between the three groups were found, Table 5.

**Table 1:** Baseline characteristics of the 290 patients

	Contaminated PF (n = 101)	Non-contaminated PF (n = 189)
Donor		
Age, years	61.3 ± 13.9	59.7 ± 15.3
Cold ischemia time, h	16.4 ± 3.8	15.8 ± 4.2
Recipient		
Age, years	55.7 ± 11.6	54.8 ± 11.5
Sex (M/F)	69/32	129/60
Induction ID, No. (%)		
Thymoglobulin	7 (7)	3 (1)
Basiliximab	100 (99)	186 (98)
Rituximab	2 (2)	0
Maintenance ID, No. (%)		
Tacrolimus	88 (87.1)	142 (75.1)
Cyclosporine	9 (8)	35 (18)
Mycophenolate mofetil	77 (76.2)	133 (70.3)
Everolimus	5 (5)	17 (9)
Azathoprine	0	3 (1)
Steroids	98 (97)	182 (96.3)

Continuous variables are expressed as mean ± SD

*PF* perfusion fluid, *ID* immunosuppression therapy

**Table 2:** Organisms isolated and frequency of their isolation in the 101 contaminated PF

Organism cultured	Incidence, No. (%)
Staphylococci	51 (50.5)
*Staphylococcus aureus*	4 (3.9)
*Staphylococcus* *epidermidis*	22 (21.7)
*Staphylococcus* *hominis*	14 (13.8)
*Staphylococcus haemolyticus*	3 (2.9)
*Staphylococcus* *warneri*	2 (1.9)
*Staphylococcus* *capitis*	2 (1.9)
*Staphylococcus* *lugdunensis*	1 (0.9)
*Staphylococcus* *simulans*	1 (0.9)
*Staphylococcus cohnii*	1 (0.9)
*Staphylococcus* *saprophyticus*	1 (0.9)
*Escherichia* *coli*	17 (16.8)
*Enterococcus* *faecalis*	6 (5.9)
*Pseudomonas* *aeruginosa*	5 (4.9)
*Acinetobacter* *baumani*	1 (0.9)
*Klebsiella* *pneumoniae*	3 (2.9)
*Candida* *albicans*	10 (9.9)
Others contaminants	19 (18.8)

Other contaminants: *Streptococcus mitis* (2), gram positive cocci (4), *Bacillus subtilis* (1), *Enterococcus raffinosus* (1), *Citrobacter freundi* (2), *Aeromonas hydrophilia* (2), *Corynebacterium* (2), *Serratia marcescensis* (1), *Morganella morganii* (1), *Klebsiella oxytoca* (2), *Enterobacter aerogenes* (1)

*PF* perfusion fluid

**Table 3:** Perioperative antibiotic prophylaxis regimens administered in recipients receiving a graft with contaminated (n = 101) and non-contaminated PF (n = 189)

Antibiotic regimen	Contaminated PF (n = 101)	Non-contaminated PF (n = 189)
	No (%)	No (%)
Co-amoxiclav	78 (77.3)	146 (77.2)
Fluoroquinolones	16 (15.8)	18 (9.5)
Other antibiotic regimens	7 (6.9)	25 (13.3)

*PF* perfusion fluid

**Table 4:** Summary of the preemptive antibiotic regimens used

Antibiotic regimen	No (%)
Fluoroquinolones	23 (44.2)
Fluconazole	9 (17.3)
Meropenem	6 (11.5)
Teicoplanin	5 (9.6)
Vancomycin	3 (5.7)
Caspofungin	2 (3.8)
Tazocin	1 (1.9)
Co-amoxiclav	1 (1.9)
Cephalosporin	1 (1.9)
Co-trimoxazole	1 (1.9)

**Table 5:** Clinical and laboratory markers of infections at the time of receiving the results of PF culture

	Group 1 (n = 52)	Group 2 (n = 28)	Group 3 (n = 21)	
Body temperature (°C)	36.2 ± 0.5	36 ± 0.5	36.1 ± 0.5	n.s.
C-reactive protein (mg/mL)	8.3 [1.5–120.8]	12.3 [0.9–43.5]	8.9 [2.5–57.00]	n.s.
Leucocyte count (cells/μL)	7402 ± 2390	6359 ± 2311	7109 ± 1899	n.s.
Time post-transplant of diagnosis of PF contamination (days)	7.1 ± 1.4	6.6 ± 0.8	7.8 ± 1.9	n.s.
Duration of PE-T (days)	11.3 ± 3.9	–	–	–

Continuous variables are presented as mean ± SD or as median [min–max], according to their distribution. The difference between groups was analyzed, respectively, with t-test or Anova and Bonferroni and with Kruskal–Wallis test. Categorical variables are presented as fraction and Pearson’s *χ* ^2 ^or, for small samples, Fisher’s exact test was employed to compare groups. Significance level for all tests was set at 0.05

*Group 1* patients with a pathogen in PF resistant to PAP that underwent PE-T, *Group 2* recipients with contaminated PF by an organism sensitive to the PAP and who did not underwent PE-T, *Group 3* recipients with a pathogen in PF resistant to PAP who did not underwent PE-T, *PF* perfusion fluid, *PAP* perioperative antibiotic prophylaxis, *PE-T* preemptive antibiotic therapy

## Discussion

Infections remain a serious complication of renal transplantation. Pathogens could be transmitted to the recipient through a number of sources including the PF used to perfuse the kidney following donor nephrectomy (Fisher et al. 2009; Sharma et al. 2005; Sauget et al. 2011; Bucholz et al. 1985). At present, with the exception of fungi contamination where there is a general consensus to treat the patient with an appropriate antifungal therapy based on PF-culture, it remains unclear whether a preemptive antibiotic therapy could lead to a reduction in the rate of infectious complications in case of PF contaminated by bacteria (Fisher et al. 2009; Matignon et al. 2008). In this retrospective study aimed to evaluate the rate of contaminated PF in our center and the impact of the preemptive therapy on the rate of PF-related infections, we found first of all a high percentage of contaminated PF together with an extremely low rate of infections PF-related. As reported by other authors (Sharma et al. 2005; Sauget et al. 2011), also in our study we found a majority of less pathogenic bacteria, such as staphylococci in positive PF. Nevertheless, a relevant number of bacteria considered highly pathogenic such as *Pseudomonas aeruginosa* and Gram-negative bacilli were found as well in our study. Consistently with the results of some recent reports, the high percentage of PF contaminated by skin contaminants such as coagulase-negative staphylococci may be due to i) the handling of the kidneys during the procurement process taking into account that all donors were multi-organ, and ii) the advances in microbiological detection that became more sensitive during the last years (Wakelin et al. 2005; Sauget et al. 2011). Until December 2011 we adopted a preemptive antibiotic treatment based on PF-culture according to Sharma et al. (2005) that suggested a preemptive therapy in the case of lactose fermenting coliform or yeast and to Fisher et al. (2009) that extended the preemptive treatment also for less virulent organisms. Starting from January 2012 to December 2012, we did not adopt a preemptive therapy in case of PF contaminated by bacteria based on the following two issues. Firstly, the results of the analysis of the data regarding the infectious complications collected in our center during January 2010 and December 2011 showed that only 1 % of the recipients who received a graft with a contaminated PF have developed an infection. Secondly, the increase of antibiotic resistant bacteria including carbapenem-resistant Gram-negative pathogens recently isolated in our hospital. Nevertheless, the inflammatory markers were screened daily until normalization together with a careful physical examination performed every day in recipients receiving a kidney with a positive PF for bacteria.

We did not find any statistical differences in the rate of infections PF-related between patients who were treated with a preemptive therapy based on the results of PF cultures and those untreated, irrespective of the isolated bacteria strain that were homogeneously distributed in the 3 groups. These findings are in agreement with recent reported data (Porco et al. 2012). Moreover, there were no differences in the following clinical parameters: leucocytes count, CRP and body temperature checked at the time of the results of the PF cultures between patients treated with preemptive therapy and those who were not.

Another important issue highlighted by this study is the reduction of the costs related to the administration of the preemptive therapy. In fact, analyzing data regarding the percentage of bacteria contaminated PF found in our study, the type (see Table 4) and the duration of the preemptive therapy (11.5 ± 4 days) adopted, we calculated that avoiding preemptive therapy in recipients who received a graft with a PF contaminated by bacteria could save about 5000 Euro per year. In addition, a relevant number of studies demonstrated that antibiotic over-prescription leads to an increased rate of morbidity and mortality from drug-resistance organisms such as extended-spectrum β-lactamases (ESBLs) and carbapenemase-producing bacteria which, in some cases, are capable of hydrolyzing almost all β-lactams including the carbapenems (Gutkind et al. 2013; Shigemura et al. 2011). Moreover, it should also be taken into account that a reduction of the use of fluoroquinolone is associated with a decrease in methicillin-resistant *Staphylococcus aureus* and a fluoroquinolone-resistant *Pseudomonas aeruginosa* isolation rate (Lafaurie et al. 2012). Thus, consistently with the results of our study, we can speculate that the rate of drug-resistance organisms might be reduced proportionally by diminishing the use of a preemptive therapy in recipients who received a graft with a contaminated PF by bacteria.

## Conclusions

At our knowledge this is the first study designed to evaluate the clinical impact of the preemptive therapy in the case of contaminated perfusion fluid. Nevertheless, we are aware of the limitations of this study. First of all this is a single center, retrospective study possibly subjected to treatment and observer biases; secondly we cannot completely exclude that the few urinary infections that developed after transplantation are due to contaminated PF even though based on the time period elapsed from transplantation to the occurrence of the infection and the antibiotic sensitivity, it is likely that the bacteria responsible of the infections are not the same isolated in the PF; thirdly the duration of the prophylactic antibiotic therapy used in our centre at the time of the study was longer compared to the accepted general practice and it could reduced the sensitivity of the urine culture.

Waiting for largest and prospective clinical trials to confirm our findings, the message emerging from this study is that although contamination of PF occurs frequently due to a variety of factors including handling of the kidney during procurement process, the rate of the PF-related infections is negligible. In addition, our data suggest that a reduction of the use of preemptive therapy, mandatory in the case of fungi isolation, could be considered in recipients with positive PF for bacteria reducing the deleterious impact of antibiotic-resistant organisms and saving money. However, we highly recommend a closely clinical and microbiologic monitoring of the recipient in case of PF contamination in order to establish a diagnosis as soon as possible and, consequently, promptly start the appropriate antibiotic therapy.

## Abbreviations

PF: perfusion fluid
PE-T: preemptive therapy
PAP: perioperative antibiotic prophylaxis
